# Evaluation of Serum Ceruloplasmin Levels as a Biomarker for Oxidative Stress in Patients With Diabetic Retinopathy

**DOI:** 10.7759/cureus.13070

**Published:** 2021-02-02

**Authors:** Gurunadh Satyanarayana, Narendra Keisham, Hitender S Batra, Subrahmanya Murti V, Mansur Khan, Sandeep Gupta, Vikram Mahindra

**Affiliations:** 1 Ophthalmology, GSL Medical College, Rajahmundry, IND; 2 Ophthalmology, Military Hospital, Jabalpur, IND; 3 Biochemistry, Military Hospital, Kirkee, IND; 4 Cardiology, Smt. Nathiba Hargovandas Lakhmichand (NHL) Municipal Medical College, Ahmedabad, IND; 5 Vitreo-Retina, Command Hospital Air Force, Bangalore, IND; 6 Cornea, Command Hospital Chandimandir, Panchkula, IND; 7 Ophthalmology, Armed Forces Medical Services, Pune, IND

**Keywords:** diabetes mellitus, diabetic retinopathy, ceruloplasmin, oxidative stress

## Abstract

Background

Elevated serum ceruloplasmin is a biomarker for oxidative stress. Diabetes mellitus (DM) is known to be a state of oxidative stress which causes complications of DM including diabetic retinopathy (DR). The role of ceruloplasmin in DR is still unclear.

Methods

Ninety patients of DM were included as cases and after evaluation sub-grouped as those with no DR, non-proliferative DR (NPDR) and proliferative DR (PDR). Serum ceruloplasmin levels were tested in all cases as well as in equal numbers of age and sex-matched controls without DM. Statistical analysis was done with p<0.05 taken as significant.

Results

Serum ceruloplasmin was significantly higher among cases as compared to controls (1222.82±306.15 IU/L versus 868.38±198.80 IU/L, p<0.01). There was no statistical difference between serum ceruloplasmin values in No DR, NPDR and PDR. On receiver operator characteristic curve (ROC) analysis for serum ceruloplasmin as a test for discriminating various parameters, it was seen that serum ceruloplasmin was a good test for discriminating DM from no DM (area under receiver operator characteristic {AUROC}=0.814, 95% CI=0.749-0.868, p<0.0001) with a cut point of >1093 IU/L yielding a sensitivity of 63.33% and specificity of 87.78%. Ceruloplasmin as a test was not found to significantly discriminate DR (total) from no DR, NPDR from no DR, PDR from no DR and PDR from NPDR.

Conclusion

Serum ceruloplasmin levels are significantly raised in patients with DM. However, serum ceruloplasmin levels do not correlate with DR severity.

## Introduction

Diabetic retinopathy (DR) is a leading cause of blindness in the world. As per data from the nationwide Indian Council of Medical Research (ICMR) - InDIAB study, 7.3% of Indians suffer from diabetes mellitus (DM) which translates to a huge burden of around 65 million people with diabetes and 77.2 million people with prediabetes [1]. Further, by 2030, India is projected to become the country with the highest burden of diabetes with about 80 million cases. As the global prevalence of diabetes increases, so will the number of people with diabetes-related complications including DR. As per a 2016 study, there are about 22 million patients with diabetic retinopathy in India [2].

Every patient with DM has the potential to develop DR over a period of time with the frequency and severity of DR being related to the duration of diabetes. Therefore, early detection of DR and its management is important to prevent disabling visual loss from DR. Several theories exist on the mechanism of diabetic complications. It has been postulated that DM is a state of chronic inflammation with increased oxidative stress. Glucose is usually metabolized by the glycolytic and tricarboxylic acid cycle pathways yielding reducing equivalents which drive the synthesis of adenosine triphosphate (ATP) by oxidative phosphorylation. However, oxidative phosphorylation also produces free radicals or reactive oxygen species (ROS), such as superoxide anion, whose production is increased by high levels of glucose [3].

Elevated levels of ROS reduce levels of nitric oxide which promotes endothelial activation, consequent leucocyte adhesion and reduced barrier function. All these changes cause microangiopathy which causes the changes observed in DR [4]. Deranged homeostasis of ROS has been implicated in the development of DR [5,6]. In this setting, various methods to quantify oxidative stress have been developed. One of the known markers of oxidative stress is ceruloplasmin. Ceruloplasmin is a circulating blue multi-copper oxidase that contains >95% of copper in the plasma. Ceruloplasmin exhibits a copper-dependent oxidase activity, which is associated with oxidation of Fe2+ (ferrous iron) into Fe3+ (ferric iron). An increase in serum ceruloplasmin levels has been reported in type 2 DM [7]. The primary physiologic role of ceruloplasmin involves plasma redox reactions. Ceruloplasmin permits the incorporation of iron into transferrin without the formation of toxic iron products. Oxygen is directly reduced to water in that redox reaction and may also represent the mechanism by which ceruloplasmin inhibits superoxide induced lipid peroxidation.

Under physiologic conditions, ceruloplasmin is also important in the control of membrane lipid oxidation, probably by direct oxidation of cations, thus preventing their catalysis of lipid peroxidation [8]. Increased ceruloplasmin levels seen in diabetes mellitus may be a protective response to an increase in circulating unbound Fe2+, which would act as a catalyst for further free radical-induced lipoperoxidation [9]. Therefore, elevated plasma ceruloplasmin levels could signal abnormally high oxidant stress. A study by Inoue et al. found that ceruloplasmin was a marker of oxidative stress in DM [10]. Therefore, this study is aimed at finding out whether serum ceruloplasmin is higher in patients of DM (with and without DR) compared to controls without DM and also whether levels of serum ceruloplasmin have any relation with the severity of DR. This may help to serve as a marker of uncontrolled DM and DR, which may help in improving their management and patient outcomes.

## Materials and methods

A prospective, observational, hospital-based, case-control study was conducted on 90 patients of type 2 DM attending eye OPD at a tertiary care hospital in western India. Another 90 age- and sex-matched individuals without DM served as controls.

A complete medical history was taken for all the cases and control subjects with an emphasis on the points that may be confounders for levels of serum ceruloplasmin. These were diabetes mellitus, hypertension, renal disease, pregnancy, oral contraceptive pills, copper toxicity, zinc deficiency, rheumatoid arthritis or any acute or chronic inflammatory disease. 

All patients underwent an ophthalmic examination and investigations. These investigations were best-corrected visual acuity by Snellen’s visual acuity chart, slit-lamp biomicroscopic examination of the cornea, anterior chamber, lens and anterior vitreous. Dilated fundus examination was done with direct ophthalmoscopy, slit-lamp bio-microscopy with 90 D lens and indirect ophthalmoscopy where required. Intraocular pressure was estimated by Goldman applanation tonometry. Systemic examination was conducted including blood pressure measurement using an electronic manometer. Optical coherence tomography (OCT) and fundus fluorescein angiography (FFA) was done in all cases of DR. The grading of DR was based on Early Treatment Diabetic Retinopathy Study (ETDRS) classification. Laboratory investigations consisted of serum ceruloplasmin level, fasting (FBS) and post-prandial blood sugar (PPBS) levels, glycosylated hemoglobin levels (HbA1c) and serum urea and creatinine levels.

Based on the clinical picture and corroborated by FFA patients with DR were grouped as Groups A, B, C and D. Group A consisted of 30 diabetics with no DR (No DR). Group B consisted of 30 diabetics with mild and moderate non-proliferative diabetic retinopathy (NPDR). Group C consisted of 30 diabetics with severe/very severe NPDR and proliferative diabetic retinopathy (PDR). These two states of DR were merged because of similar management strategies. Groups A, B and C together form the study group of 90 patients. Group D consisted of 90 age- and sex-matched controls. 

Patients of type 2 diabetes mellitus attending Eye OPD were included in the study. Patients unwilling to give consent were excluded along with patients with coexisting diabetic nephropathy, patients with Wilson’s/ Menke’s Disease, patients with any other systemic acute or chronic inflammatory disease, patients having retinopathy other than DR, 

Serum ceruloplasmin measurement was done by the Somani and Ambade method. Ceruloplasmin is a copper-containing alpha 2 glycoprotein which possesses significant oxidase activity against ferrous ions. Chromogen (0.5 mmol/L) solution (Reagent-1): was made by dissolving 159.65 mg of norfloxacin in 1000 mL of acetate buffer (0.45 mol/L, pH 5.4) containing 0.2% Triton X-100. Substrate (2.04 mmol/L) solution (Reagent-2): was made by sequentially dissolving, 320 mg of dithiothreitol (DTT) and 800 mg of ferrous ammonium sulfate, Fe(NH4)2(SO4)2·6H2O, in 1000 mL of distilled water. Both Reagent-1 and Reagent-2 were stable for more than 6 months at 4 °C as well as at room temperature. Standard (6.0 mmol/L): was made by dissolving 2.896 g of ammonium iron (III) sulfate dodecahydrate, NH4Fe(SO4)2·12H2O, in 1000 mL of acetic acid (0.20 mmol/L). Serum sample (50 μL) was added to 1000 μL of Reagent-1 and mixed. After a 1-min interval, 150 μL of Reagent-2 was added. The mixture was aspirated into Shimadzu CL-750 spectrophotometer with the following settings: measuring wavelength 377 nm, lag period 10 s, kinetics time 30 s, temperature 37°C, aspiration volume 1 mL and factor 2012. For the blank correction, a blank was run using distilled water in place of the serum sample.

Statistical analysis

There were a total of 90 subjects and 90 controls. For comparison of means between groups, unpaired t-tests and analysis of variance (ANOVA) were used. IBM Statistical Package for the Social Sciences (SPSS) Package 16.0 (IBM, Armonk, NY) was used for analysis.

## Results

The mean age of the cases was 59.74 ± 6.77 years which was well matched to controls. As expected, the mean fasting blood sugar (FBS), post-prandial blood sugar (PPBS) and HbA1C were significantly higher in cases as compared to controls. There was no statistical difference in serum values of urea and creatinine among cases and controls. Serum ceruloplasmin was significantly higher among cases (with DM) as compared to controls (1222.82 ± 306.15 IU/L versus 868.38 ± 198.80 IU/L, p < 0.01) (Table 1).

**Table 1:** Comparison of cases and controls DM: diabetes mellitus; HbA1C: glycosylated hemoglobin; FBS: fasting blood sugar; PPBS: post-prandial blood sugar

Parameter	Cases (patients with DM)	Controls (without DM)	P-value
Age (years)	59.74 ± 6.77	58.22 ± 7.95	0.129
FBS (mg/dL)	129.14 ± 15.65	84.93 ± 9.80	<0.001
PPBS (mg/dL)	219.70 ± 34.35	128.26 ± 15.71	<0.001
Serum Urea (mg/dL)	25.93 ± 6.77	23.62 ± 6.22	0.016
Serum Creatinine (mg/dL)	0.87 ± 0.16	0.89 ± 0.17	0.412
Serum Cerulopasmin (IU/L)	1222.82 ± 306.15	868.38 ± 198.80	<0.01
HbA1C	8.84 ± 1.28	5.94 ± 0.93	<0.01

Of a total 90 cases of DM, subdivision was done as those without DR (no DR = 30 patients), with non-proliferative DR (NPDR = 30 patients) and those with proliferative DR (PDR = 30 patients). Comparisons among these subgroups are shown in Table 2. HbA1C was significantly lower in NPDR as compared to No DR (8.65 ± 1.28 versus 9.41 ± 1.11, p<0.05) and in PDR as compared to No DR (8.45 ± 1.28 versus 9.41 ± 1.11, p<0.05). There was no statistical difference between HbA1C values in PDR and NPDR. Further, there was no statistical difference between serum ceruloplasmin values in No DR, NPDR and PDR (Table 2).

**Table 2:** Distribution of cases with comparisons among the subgroups NPDR: non-proliferative diabetic retinopathy; PDR: proliferative diabetic retinopathy; FBS: fasting blood sugar; PPBS: post-prandial blood sugar; HbA1C: glycosylated hemoglobin

Parameter	Diabetes mellitus without retinopathy (No DR)	NPDR	PDR	P-value (no DR versus NPDR)	P-value (no DR versus PDR)	P-value (NPDR versus PDR)
Age (years)	58.77 ± 8.15	57.87 ± 5.37	62.60 ± 5.67	0.594	0.025	0.006
FBS	123.37 ± 9.53	128.40 ± 10.42	135.67 ± 21.76	0.196	0.002	0.064
PPBS	209.83 ± 23.34	219.93 ± 25.05	229.33 ± 47.45	0.250	0.028	0.284
Serum Urea (mg/dL)	23.70 ± 5.83	25.93 ± 5.99	28.17 ± 7.77	0.193	0.010	0.193
Serum Creatinine (mg/dL)	0.84 ± 0.17	0.83 ± 0.16	0.93 ± 0.13	0.740	0.022	0.009
Serum Ceruloplasmin (IU/L)	1167.60 ± 295.69	1238.33 ± 287.40	1262.53 ± 335.53	0.375	0.234	0.761
HbA1C	9.41 ± 1.11	8.65 ± 1.28	8.45 ± 1.28	0.018	0.003	0.529

**Figure 1:**
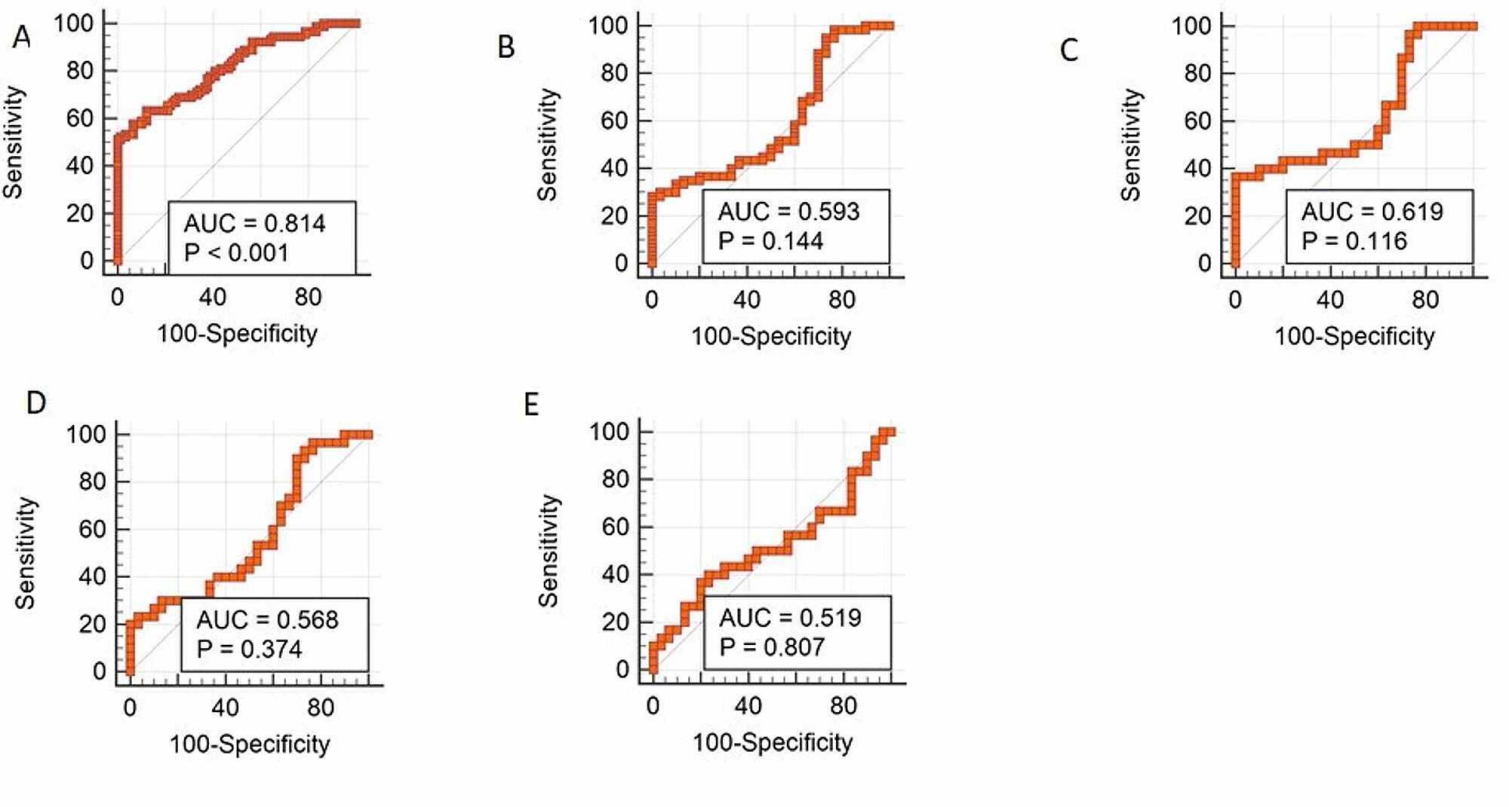
Area under receiver operator characteristic (AUROC) curves for ceruloplasmin (A) DM versus No DM; (B) DR versus No DR; (C) PDR versus No DR; (D) NPDR versus No DR; (E) PDR versus NPDR DM: diabetes mellitus; DR: diabetic retinopathy; PDR: proliferative diabetic retinopathy; NPDR: non-proliferative diabetic retinopathy

On receiver operator characteristic (ROC) curve analysis for serum ceruloplasmin as a test for discriminating various parameters, it was seen that serum ceruloplasmin was a good test for discriminating DM from no DM (area under ROC curve =0.814, 95% CI=0.749-0.868, p<0.0001) with a cut point of >1093 IU/L yielding a sensitivity of 63.33% and specificity of 87.78%. Ceruloplasmin as a test was not found to significantly discriminate DR (total) from no DR, NPDR from no DR, PDR from no DR and PDR from NPDR (Figure 1).

## Discussion

In the present study, serum ceruloplasmin was significantly higher in patients with DM (cases) as compared to controls (without DM). Existing literature has also shown higher levels of ceruloplasmin in patients with DM. In the largest data on ceruloplasmin in patients with DM from Japan, serum ceruloplasmin was significantly higher in people with DM compared to those without [9]. The same conclusion was also drawn from a recent study from Nepal, which also showed a correlation of higher low-density cholesterol (LDL) to higher ceruloplasmin levels. These findings suggest that DM is a state of increased oxidative stress [11].

In the present study, there was no statistical difference in serum levels of ceruloplasmin among diabetic patients without retinopathy as compared to those with DR. Further, among patients of DR, there was no significant difference in ceruloplasmin levels among patients with proliferative DR versus non-proliferative DR. This suggests that ceruloplasmin levels may not increase with increasing retinopathy and thus may not help to discriminate the severity of retinopathy. The role of serum ceruloplasmin levels in diabetic complications has been investigated earlier with conflicting results. Oxidative stress has been linked to retinopathy in DM with evidence from animal models available [12]. In a study by Mohora et al. on patients with diabetic foot, patients with retinopathy had significantly higher levels of ceruloplasmin compared to those without [13]. The same study also found a similar trend in diabetic nephropathy and neuropathy wherein there was no significant positive trend in serum ceruloplasmin levels with the severity of the diabetic complication. In a study by Nowak et al., 41 patients with type 1 DM were examined. Interestingly, that study showed that ceruloplasmin levels were lower in those with DR than those without [14]. However, continuing the debate on oxidative stress, in contrast to other studies, a Korean study on diabetic nephropathy found that higher ceruloplasmin levels were associated with faster progression of nephropathy [15].

In the present study, serum HbA1C was significantly lower in NPDR as compared to No DR and in PDR as compared to No DR. There was no statistical difference between HbA1C values in PDR and NPDR. These findings are contrary to the general appreciation that diabetic complications are associated with poor glycemic control. Similar results were seen in a large Chinese study wherein there was no significant positive correlation between retinopathy grade and HbA1C [16]. These may be due to two reasons - patients with PDR and NPDR may have a confounding bias of stricter treatment and onset of retinopathy may have other factors rather than being solely dependent on glycemic status alone. One of the important factors is the presence of coexistent nephropathy. Various studies have found that in the presence of nephropathy, HbA1C had a less accurate correlation with glycemic control [17]. The 'metabolic memory' theory, as described in the results of many pivotal trials shows that early intensive glycemic control reduces the onset of complications of diabetes [18]. This effect may also confound correlations of the glycemic control with the severity of complications in DM.

In the present study, using ROC analysis, it was found that ceruloplasmin as a test was good in discriminating only DM from no DM and was not useful in discriminating DR from no DR. A ceruloplasmin level >1093 IU/L yielded a sensitivity of 63.33% and specificity of 87.78% for diagnosing DM (area under receiver operator characteristic {AUROC}=0.814, p<0.0001). Similar results were obtained by Sharma et al. wherein a cutoff of 46.5 mg/dL of serum ceruloplasmin had a sensitivity of 87.5% and specificity of 62% (AUROC=0.881, p<0.01) for discriminating those with DM compared to those without [11]. These results, along with those described above suggest that serum ceruloplasmin is definitely elevated in subjects with DM establishing DM as a state of increased oxidative stress. However, the relationship of serum ceruloplasmin with onset, progression and severity of diabetic complications is less well established and requires further evaluation in larger studies.

Whether treatment with antioxidants has a role in DM is a subject of open debate. Antioxidants are of some benefit in experimental animals with diabetic retinopathy. However, this has not translated into clinical trials with some small uncontrolled clinical trials showing no benefit over placebo [3]. Classical clinical trials like the Diabetes Control and Complications Trial (DCCT) drive home the importance of good glycemic control. In fact, some of the diagnostic cut-offs for DM were derived from data on trends of incidence of retinopathy with rising sugar levels in a classical trial by Dorf et al. on the Pima Indian population in the Amazonian areas, an area with a known higher incidence of DM [19]. These emphasize the fact that the onus still remains on good glycemic control which is the cornerstone of the management of DM.

Study limitations

The major limitation of the present study is that the sample size is small. This makes it difficult to draw definitive conclusions about the role of ceruloplasmin levels in DR.

## Conclusions

This study started with the question of the role of serum ceruloplasmin levels in DM as also in the main ocular complication of DM i.e., DR. This study has shown that serum values of ceruloplasmin are significantly elevated in patients with DM when compared to controls without DM. However, among patients of DM, there was no statistical difference in levels of serum ceruloplasmin among those with DR compared to those without DR. Further, among patients with DR, there was no statistical difference in serum ceruloplasmin levels between those with NPDR compared to those with PDR. Higher ceruloplasmin levels in patients with DM as shown by this study, adds to the already existing body of evidence that DM is a state of increased oxidative stress. However, the reasoning that levels of serum ceruloplasmin would rise with increasing severity of retinopathy because of the presumed increase in anti-oxidant activity was not corroborated by this study. 
